# Differential Effects of Lycium Barbarum Polysaccharides and Seaweed Polysaccharides on Growth, Immunity, and Hepatic Lipid Metabolism in Juvenile Rainbow Trout (*Oncorhynchus mykiss*)

**DOI:** 10.3390/antiox15101314

**Published:** 2026-10-12

**Authors:** Bingchen Guo, Jing Wang, Ran Zhao, Shaowu Li, Di Wang

**Affiliations:** 1 Key Laboratory of Aquatic Animal Diseases and Immune Technology of Heilongjiang Province, Heilongjiang River Fisheries Research Institute, Chinese Academy of Fishery Sciences, Harbin 150070, China; gbcbc0528@163.com (B.G.); wangjing@hrfri.ac.cn (J.W.); zhaoran@hrfri.ac.cn (R.Z.); lishaowu@hrfri.ac.cn (S.L.); 2 Laboratory of Fish Physiology and Resource Utilization, College of Life Science and Technology, Harbin Normal University, Harbin 150025, China

**Keywords:** *Oncorhynchus mykiss*, Lycium barbarum polysaccharides, Seaweed polysaccharides, growth, metabolism, immunity

## Abstract

Polysaccharides play crucial roles in medicine and nutrition, serving as dietary additives that help maintain fish health. This study systematically evaluated the effects of dietary supplementation with plant-derived Lycium barbarum polysaccharides (LBP) and Seaweed-derived polysaccharides (SPs) on growth, metabolism, and immune function in rainbow trout (*Oncorhynchus mykiss*). A total of 270 juvenile rainbow trout (15.50 ± 0.71 g) were randomly divided into three groups (control group, CG; 1.5 g/kg LBP; 1.5 g/kg SPs), with 90 fish in each group. The feeding trial lasted 56 days, with sampling at days 28 and 56. Compared with CG, SPs increased WGR by approximately 39.4%, decreased FCR by approximately 44.7%, and elevated muscle Ala, Glu, and Gly contents. SPs also improved intestinal microbiota balance and promoted the synthesis of amino acids and short-chain fatty acids (SCFAs). In addition, SPs significantly enhanced CAT and SOD activities and upregulated *IL-10* expression by approximately 2.3-fold. Compared with the CG, LBP can reduce the total cholesterol content by 33.3%, inhibit lipid synthesis, and promote fatty acid oxidation. It also upregulates the expression of the *PPARα* gene by approximately 2.4-fold. In the *Aeromonas salmonicida* challenge test, survival rates of the SPs and LBP groups were 30% and 20% higher, respectively, than that of CG. These results indicate that SPs were more effective in promoting growth and enhancing immunity, whereas LBP showed superior performance in metabolic regulation. This study provides a basis for the targeted application of these two substances.

## 1. Introduction

The aquaculture industry has grown substantially in recent decades due to the increasing global demand for sustainable protein sources [1]. Rainbow trout (*Oncorhynchus mykiss*) is highly favored for its rapid growth, strong adaptability, delicious flesh, and rich nutritional value [2]. Rainbow trout is one of the most widely farmed cold-water fish species in China. Although rainbow trout is a freshwater fish, it has a relatively strong salt tolerance and can be domesticated to adapt to seawater [3]. The impact of salinity on its growth and physiology depends on the size of the fish and the salinity level [4]. Studies have indicated that marine farming may enhance the growth performance, disease resistance, and flavor of rainbow trout [5]. Currently, the cross-farming of rainbow trout between land and sea is developing rapidly. In high-density intensive farming, it often leads to deterioration of water quality and increases stress on fish. This consequently leads to fish contracting various diseases, thereby hindering the healthy development of the industry [6,7]. To address this situation, the aquaculture sector began employing antibiotics to prevent and treat diseases. However, their misuse has led to significant issues such as increased bacterial resistance, drug residues, and ecological damage [8,9]. Consequently, developing safe, effective, and environmentally friendly alternatives to antibiotics has become a critical research focus in aquaculture [10]. In recent years, plant polysaccharides have gained growing attention as promising alternatives [11,12,13,14].

They have been reported to exhibit multiple biological activities, including promoting growth, regulating immunity, and influencing metabolism [11,15,16]. In terms of growth, polysaccharides can enhance gut health, promote the activities of digestive enzymes such as proteases, amylases, and lipases, and improve the efficiency of nutrient absorption and utilization. These digestive enzyme activities are typically expressed as specific activity (U/mg protein) in fish nutrition studies. This ultimately enhances key growth metrics and the expression of associated genes in fish, thereby accelerating growth [17,18]. In terms of metabolism, polysaccharides participate in regulating glucose and lipid metabolism. This aids in maintaining metabolic homeostasis within the organism [11]. Elevated muscle levels of polyunsaturated fatty acids (notably EPA and DHA) and essential amino acids were also observed [19]. Consequently, this improves the quality and nutritional value of the fish meat. In terms of immune regulation, several polysaccharides have been shown to act as potent immunostimulants in fish, including plant-derived polysaccharides such as APS [12], and LBP [11], as well as seaweed-derived polysaccharides such as fucoidan and laminarin [20]. Their core mechanism lies in activating the non-specific immune system of fish. This manifests through stimulating the proliferation and activity of immune cells such as macrophages, neutrophils, and lymphocytes, promoting the secretion of immune factors such as lysozyme, complement proteins, and cytokines (e.g., *IL-1β*, *TNF-α*, and *IL-10*), and elevating the levels of immune molecules such as immunoglobulin M (IgM) and antimicrobial peptides. Consequently, this enhances the organism’s disease resistance [21,22].

At present, numerous studies have been conducted by domestic and international scholars on the application of polysaccharides in various aquatic animals. Such as *Cyprinus carpio* [23], *Micropterus salmoides* [24] and *Oreochromis niloticus* [25], and have achieved remarkable and positive progress. In salmonid fish, some studies also indicate that polysaccharides have potential promoting effects on their growth and health [26,27]. Previous studies have provided valuable evidence for the effects of single polysaccharides. However, due to the differences in species, doses, periods and experimental conditions among these studies, direct comparisons between different polysaccharides are rather challenging. Therefore, in this study, Lycium barbarum polysaccharides (LBP) and Seaweed polysaccharides (SPs) were evaluated in parallel within the same experimental system. This study selected LBP and SPs based on the complementary functional characteristics reported in previous aquatic research. LBP has been reported to regulate lipid metabolism by activating PPARα pathway genes, inhibiting lipid synthesis, and reducing hepatic lipid accumulation in aquatic animals [25]. In contrast, SPs can promote weight gain in various fish species and optimize the gut microbiota. They can also enhance antioxidant and anti-inflammatory capacities, thereby improving growth and immune function. LBP and SPs have distinct functional advantages. SPs primarily focus on growth promotion and immune enhancement, whereas LBP excels in regulating lipid metabolism. Therefore, we hypothesized that their combined use could exert synergistic effects in rainbow trout. To test this hypothesis, the present study separately evaluated the effects of dietary supplementation with LBP and SPs alone in rainbow trout. These effects were assessed in terms of growth, gut microbiota, antioxidant status, immune response, and hepatic lipid metabolism. This work lays the foundation for the future development of combined feed additives containing both.

## 2. Materials and Methods

### 2.1. Animal Ethics Statement

All animal experiments were approved by the Animal Care and Use Committee of the Heilongjiang River Fisheries Research Institute, Chinese Academy of Fishery Sciences, and were conducted in accordance with its ethical guidelines. Approval code: 20250506-001.

### 2.2. Experimental Diet and Animal Feeding

Clinically healthy juvenile rainbow trout (*Oncorhynchus mykiss*) of uniform size (initial body weight 15.50 ± 0.71 g) were obtained from Benxi, Liaoning Province and reared in a flow-through system at the Heilongjiang River Fisheries Research Institute, Chinese Academy of Fishery Sciences. Before the experiment, fish were acclimated for 7 days under the same environmental conditions. All the fish showed no clinical symptoms and their feeding and swimming behaviors were normal [28,29]. A total of 270 rainbow trout were randomly divided into three groups, with 90 fish in each group. Before the feeding experiment began, the basic feed was acclimated for 7 days. Fish were fed twice daily (08:30 and 16:30) at 1–1.5% of body weight for 56 days, with the feeding amount adjusted every two weeks. A 56-day period was chosen as it is commonly used in juvenile rainbow trout nutritional studies and allows sufficient time to detect treatment effects on growth performance, feed utilization, and metabolic and immune indicators [11,30,31].

Lycium barbarum polysaccharides (LBP, S31394) and Seaweed polysaccharides (SPs, S27817) were obtained from Shanghai Yuanye Bio-Technology Co., Ltd. (Shanghai, China). LBP is a water-soluble protein polysaccharide extracted from wolfberry. It is composed of arabinose, glucose, galactose, mannose, xylose and rhamnose, with a purity of ≥50% (UV). SPs are a kelp polysaccharide from Laminaria digitata, which is a β-1,3-glucan with β-1,6 branches, and its purity is 70% (BR). The basal diet was obtained from Shandong Hanye Bio-Technology Co., Ltd., with the main ingredients including fish meal, full-fat soybean, wheat flour, calcium phosphate dibasic, refined fish oil, lysine, methionine, vitamin premix, and trace mineral premix. The nutritional composition of the basal diet was as follows: crude protein ≥ 44%, crude fat ≥ 10%, crude fiber ≤ 5%, crude ash ≤ 14%, calcium 1.0–3.0%, total phosphorus ≥ 0.9%, moisture ≤ 10%, and lysine ≥ 2.6%. The basal diet served as the control group (CG). Based on the basal diet, 1.5 g/kg LBP and SPs were added as experimental groups. Previous studies have demonstrated that various plant polysaccharides, when supplemented at a dosage of 1–2 g/kg of feed, can effectively enhance the growth performance, immune capacity, and metabolic regulation of aquatic animals [11,23,32]. The basal diet was a commercial feed, and the additives were supplemented as described previously. Briefly, the basal diet was ground, sieved, and accurately weighed, then mixed with the LBP and SPs additives. To ensure homogeneous mixing, 1 L of sterile water was added according to the specified dosage, and the mixture was thoroughly blended. The mixture was then extruded into pellets of 3 mm diameter, dried at 60 °C, sealed in sterile bags, and stored at −20 °C until use. The same procedure was applied to the basal diet of the CG, except that no LBP and SPs were added [11,30,31].

### 2.3. Calculation and Analysis of Growth Performance Indicators

The calculation formula for growth performance parameters is as follows:

Weight gain rate (WGR, %) = (W_F_ − W_I_)/W_I_ × 100%

Specific growth rate (SGR, %) = (Ln W_F_ − Ln W_I_)/56 × 100%

Feed conversion ratio (FCR) = Total feed consumption/(W_F_ − W_I_)

Hepatosomatic index (HSI, %) = (W_L_/W_W_) × 100%

Viscerosomatic index (VSI, %) = (W_V_/W_W_) × 100%

Condition Factor (CF, g/cm^3^) = W_F_/L_B_^3^

Abdominal fat percentage (AFP, %) = W_A_/W_F_ × 100%

Among them, W_I_ (g), W_F_ (g), W_W_ (g), W_V_ (g), W_L_ (g), W_A_ (g) and L_B_ (cm) respectively represent the initial weight, final weight, whole fish weight, internal organ weight, liver weight, abdominal fat, and body length of the fish.

### 2.4. Sample Collection

Following the conclusion of the 56-day feeding experiment, tissue samples were collected after a 24-h fast. Nine fish per tank were collected at random and anesthetized using MS222 (75 mg/L) [33,34,35]. After collecting the livers, muscles, intestines and intestinal contents of 6 fish, the tissues were rapidly frozen with liquid nitrogen and stored at −80 °C for subsequent biochemical analysis. The tissues of the remaining 3 fish were used for histological examination.

### 2.5. Determination and Analysis of Amino Acids and Fatty Acids in Muscle Tissue

Muscle tissue samples were sent to Hubei Anyi Biotechnology Co., Ltd. (Wuhan, China). for the following analyses. Muscle hydrolyzed amino acids were determined according to the GB/T 5009.124-2016 [36] method. Approximately 10–20 mg of muscle sample was weighed and subjected to microwave-assisted acid hydrolysis pretreatment. An automatic amino acid analyzer (S-433D, Sykam, Eresing, Germany) was used to determine the composition and content of amino acids. For fatty acids, methyl esters were prepared via transesterification according to a previous method [37], and their composition was determined by gas chromatography.

### 2.6. Biochemical Analysis

Liver tissue samples (roughly 0.1 g) were processed in 1 mL of phosphate-buffered saline (PBS, 0.01 M, pH 7.4) using a tissue homogenizer. After homogenization, the mixture was subjected to centrifugation at 13,800× *g* for 10 min at 4 °C using a benchtop high-speed micro-refrigerated centrifuge (Scilogex, Beijing, China). The resulting supernatant was then harvested for subsequent enzyme activity analyses. The activities or contents of hepatic glutathione peroxidase (GSH-Px), catalase (CAT), superoxide dismutase (SOD), malondialdehyde (MDA), alanine aminotransferase (ALT), aspartate aminotransferase (AST), total cholesterol (TC), triglyceride (TG), hepatic lipase (HL), pyruvate kinase (PK), hexokinase (HK), and phosphofructokinase (PFK) were determined using commercial assay kits (Boxbio Science & Technology, Beijing, China). The key assay parameters were as follows: GSH-Px was assayed at 37 °C; SOD, CAT, PK, HK, PFK, AST and ALT were assayed at 25 °C; one unit of enzyme activity was defined as the amount of enzyme that catalyzes the conversion of 1 μmol of substrate per minute under the assay conditions.

### 2.7. Histological Staining

The liver and intestinal fixed samples were processed using histological techniques. Intestinal tissues were fixed in Bouin’s solution for 24–48 h, rinsed under running water, and dehydrated through a graded ethanol series (70–100% II, 45–60 min each). After clearing in xylene (30–60 min), tissues were infiltrated with paraffin via a 1:1 paraffin-xylene mixture, then pure paraffin I and II (1–2 h each at 58–62 °C). Specimens were embedded in molten paraffin (60–62 °C) using pre-warmed forceps, with cut surfaces facing downward and centered in molds. Solidified blocks were chilled in cold water or a cooling plate [38]. Sections (4 μm) were cut on a Leica RM 2016 microtome, stained with H&E, and examined under a Leica microscope for image acquisition and data archiving. After fixation in 4% paraformaldehyde for ≥48 h, liver tissues were dehydrated, embedded in OCT, and sectioned at 8–10 µm. Sections were stained with Oil Red O for 15 min in the dark, then sealed with glycerin gelatin [39].

### 2.8. Quantitative Real-Time PCR Analysis

Total RNA was extracted from rainbow trout liver using Trizol (Takara, Dalian, China), and its quality and concentration were assessed by agarose gel electrophoresis and NanoDrop 2000 (Thermo, Guangzhou, China). RNA was reverse-transcribed to cDNA using StarScript Pro kit (GenStar, Suzhou, China). qPCR was performed with TB Green^®^ Premix Ex Taq™ II (Takara, Dalian, China) on a StepOnePlus™ system (Software v2.2). Each 20 μL reaction contained: 10 μL of 2 × TB Green premix, 1 μL of forward and reverse primers (each at 10 μmol/L), 2 μL of cDNA template, and 6 μL of ddH_2_O. Cycling: 95 °C 30 s; 40 cycles of 95 °C 5 s, 60 °C 34 s; then melt curve [40]. Perform gradient dilution on cDNA to obtain Ct values; use lg (dilution factor) as X and Ct as Y to conduct linear regression: Y = KX + b. The primer efficiency (E) is calculated from the slope of the standard curve, using the formula: E = 10^(−1/k)^ − 1, and the amplification efficiency (%) = E × 100%, with the ideal value being 100%; 90% to 110% [38]. *β-actin* and *GAPDH* were used as internal reference genes, and the relative expression levels were calculated using the 2^−ΔΔCt^ method. The primer sequences are listed in Table 1. The primer sequences were based on previously verified sequences and were synthesized by Heilongjiang Genesoul Technology Technology Co., Ltd. (Harbin, China).

### 2.9. Intestinal Flora Analysis and Non-Targeted Metabolomics Analysis of Intestinal Contents

Following the conclusion of the 56-day feeding experiment, intestinal contents were collected after a 24-h fast. For each group, the intestinal contents of six fish were processed individually and subjected to 16S rRNA high-throughput sequencing on the Illumina platform (Personalbio, Shanghai, China). Total DNA was extracted using the Tiangen Bacterial DNA Kit and quality-assessed by 0.8% agarose gel electrophoresis and UV spectrophotometry. The 16S rRNA V3-V4 region was PCR-amplified with primers F (ACTCCTACGGGAGGCAGCA) and R (GGACTACHVGGGTWTCTAAT) using NEB Q5 polymerase at 98 °C for 2 min, 30 cycles of 98 °C for 15 s, 55 °C for 30 s, 72 °C for 30 s, and 72 °C for 5 min. Products were verified on 2% agarose gels, gel-purified, and quantified. Libraries were prepared with the Illumina TruSeq Nano DNA LT kit (end-repair, A-tailing, adapter ligation), purified twice with AMPure XP beads, and size-selected; quality and concentration were checked via Agilent Bioanalyzer and Promega QuantiFluor (qualified libraries ≥ 2 nM) [55]. Paired-end 2×300 bp sequencing was performed on an Illumina MiSeq platform (MiSeq Reagent Kit V3) by Personalbio (Shanghai). The raw sequencing data have been submitted to the NCBI database, with the accession number PRJNA1517957.

### 2.10. Bacterial Challenge

After the 56-day feeding trial, 20 fish per group were randomly selected for the challenge test. Fish were injected intramuscularly with 1 × 10^7^ CFU/mL *Aeromonas salmonicida*, and clinical signs and mortality were monitored for 14 days. Cumulative survival was analyzed by the Kaplan–Meier method and compared using the log-rank (Mantel–Cox) test. The *A. salmonicida* strain AS80 was isolated from diseased rainbow trout and confirmed to be pathogenic [56].

### 2.11. Data Processing and Statistical Analysis

Data are presented as mean ± SEM. Statistical analyses were performed using SPSS 27.0.1. Intergroup differences were evaluated by one-way ANOVA followed by Duncan’s post hoc test, with significance set at *p* < 0.05. Bacterial survival rates were analyzed by the Kaplan–Meier method, and survival curves were compared using the log-rank (Mantel–Cox) test. Graphs were created with GraphPad Prism 10.0.

## 3. Results

### 3.1. Fish Growth Performance

Table 2 shows that the SPs group had significantly higher WGR and SGR than the CG and LBP groups, increasing by 39.4% and 18.7 respectively (*p* < 0.05), and lower FCR than the CG, decreasing by 44.7% (*p* < 0.05). The LBP group showed significantly lower VSI than the CG (*p* < 0.05). HSI was significantly lower in the SPs group than in the CG (*p* < 0.05), with a similar trend in the LBP group. SI was significantly higher in the SPs group than in the other two groups (*p* < 0.05). AFP was significantly lower in the LBP group than in the CG and SPs groups (*p* < 0.05).

### 3.2. Composition of Muscle Amino Acids and Fatty Acids

**Figure 1:**
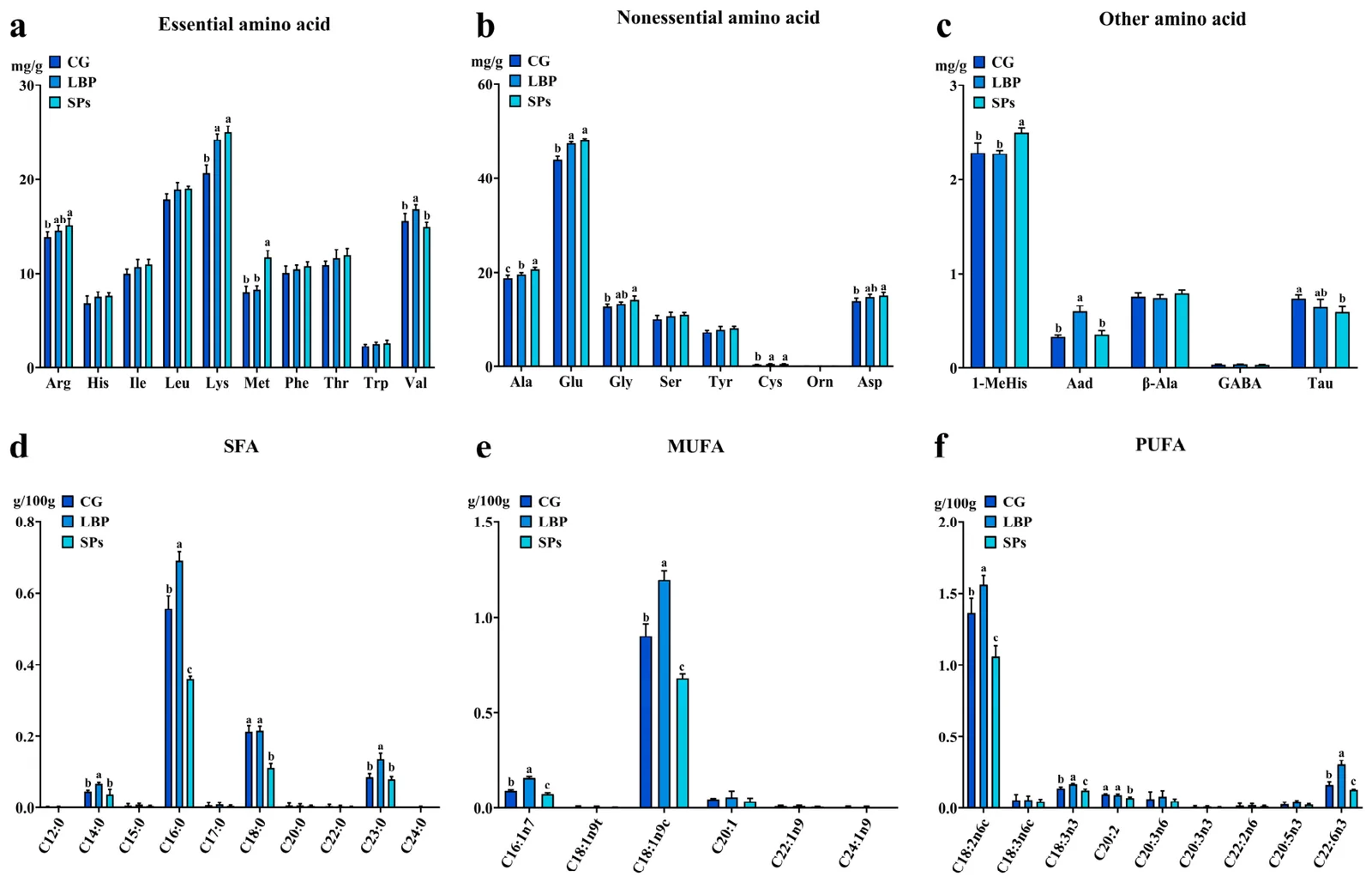
Effect of polysaccharides on amino acids in rainbow trout muscle tissue. (**a**–**c**): amino acids. (**a**) Essential amino acid; (**b**) Nonessential amino acid; (**c**) Other amino acids; d-e: fatty acids. (**d**) SFA; (**e**) MUFA; (**f**) PUFA. Note: Values (mean ± SEM) in the same row with different superscripts are substantially different (*p* < 0.05). The unit for amino acids is mg/g, and the unit for fatty acids is g/100g.

This study analyzed amino acid and fatty acid compositions of the CG, LBP, and SPs groups. Amino acid results (Figure 1a–c) showed that essential amino acids Met, Phe, and Arg were significantly higher in the LBP and SPs groups than in the CG (*p* < 0.05, Figure 1a). Val was significantly lower in the SPs group than in the CG and LBP groups (*p* < 0.05), with no significant difference between CG and LBP. Among non-essential amino acids, Ala was significantly higher in the LBP and SPs groups than in the CG (*p* < 0.05, Figure 1b). For other functional amino acids, Aad was significantly lower in the LBP and SPs groups than in the CG (*p* < 0.05, Figure 1c).

Fatty acid results (Figure 1d–f) showed that among saturated fatty acids, C16:0 was significantly higher in the LBP group than in the CG and SPs groups (*p* < 0.05, Figure 1d). For monounsaturated C18:1n9c, the LBP group had the highest content, significantly higher than both the CG and SPs groups (*p* < 0.05, Figure 1e). Polyunsaturated C18:2n6c and C22:6n3 were also significantly higher in the LBP group than in the CG and SPs groups (*p* < 0.05, Figure 1f), whereas most polyunsaturated fatty acids in the SPs group were lower than those in the CG.

### 3.3. Histological Analysis

**Figure 2:**
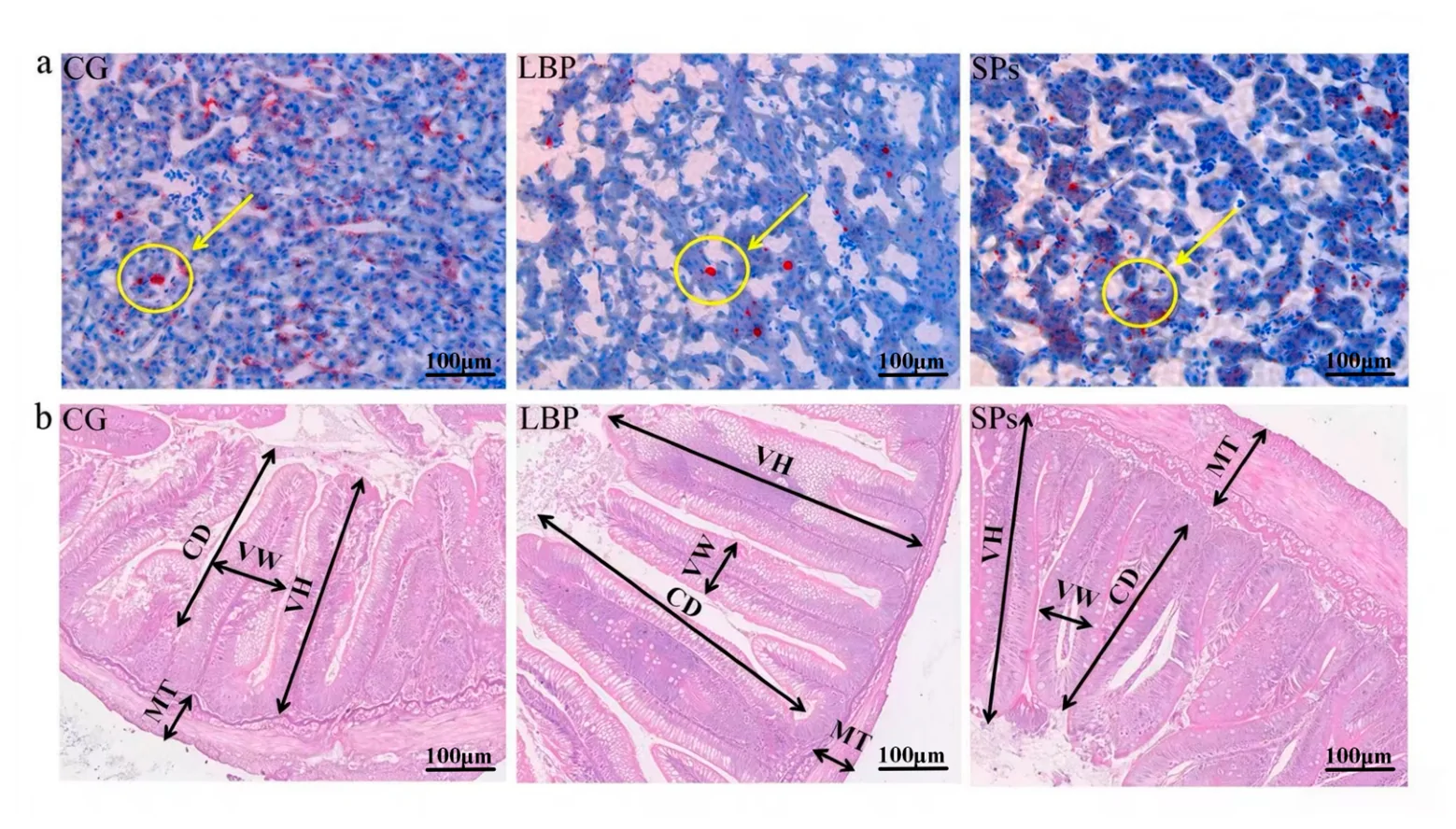
Effects of polysaccharides on lipid deposition and intestinal morphology in rainbow trout (**a**) liver oil red O staining; (**b**) intestinal H&E staining. Among them, VH: Villus height; VW: Villus width; CD: Crypt depth; MT: Muscle thickness. Scale bar: 100 µm. The yellow arrows and circles represent lipid droplets.

Oil red O staining (Figure 2a) showed numerous dispersed red lipid droplets in the CG’s liver. The LBP group had substantially fewer droplets, with only a small amount. The SPs group showed an intermediate number. H&E staining (Figure 2b) showed loose intestinal villi in the CG, while both the LBP and SPs groups had tighter villi arrangement and significantly better VH, VW, CD, and MT than the CG. The SPs group exhibited the best intestinal structural integrity and development, with higher villus height, deeper crypts, and thicker muscularis, indicating that both LBP and SPs improve intestinal morphology, with SPs being more effective. Table 3 further confirms that VH, VW, CD, and MT were significantly higher in the SPs group than in both the CG and LBP groups (*p* < 0.05).

### 3.4. Antioxidant Capacity and Metabolic Enzyme Activities

**Figure 3:**
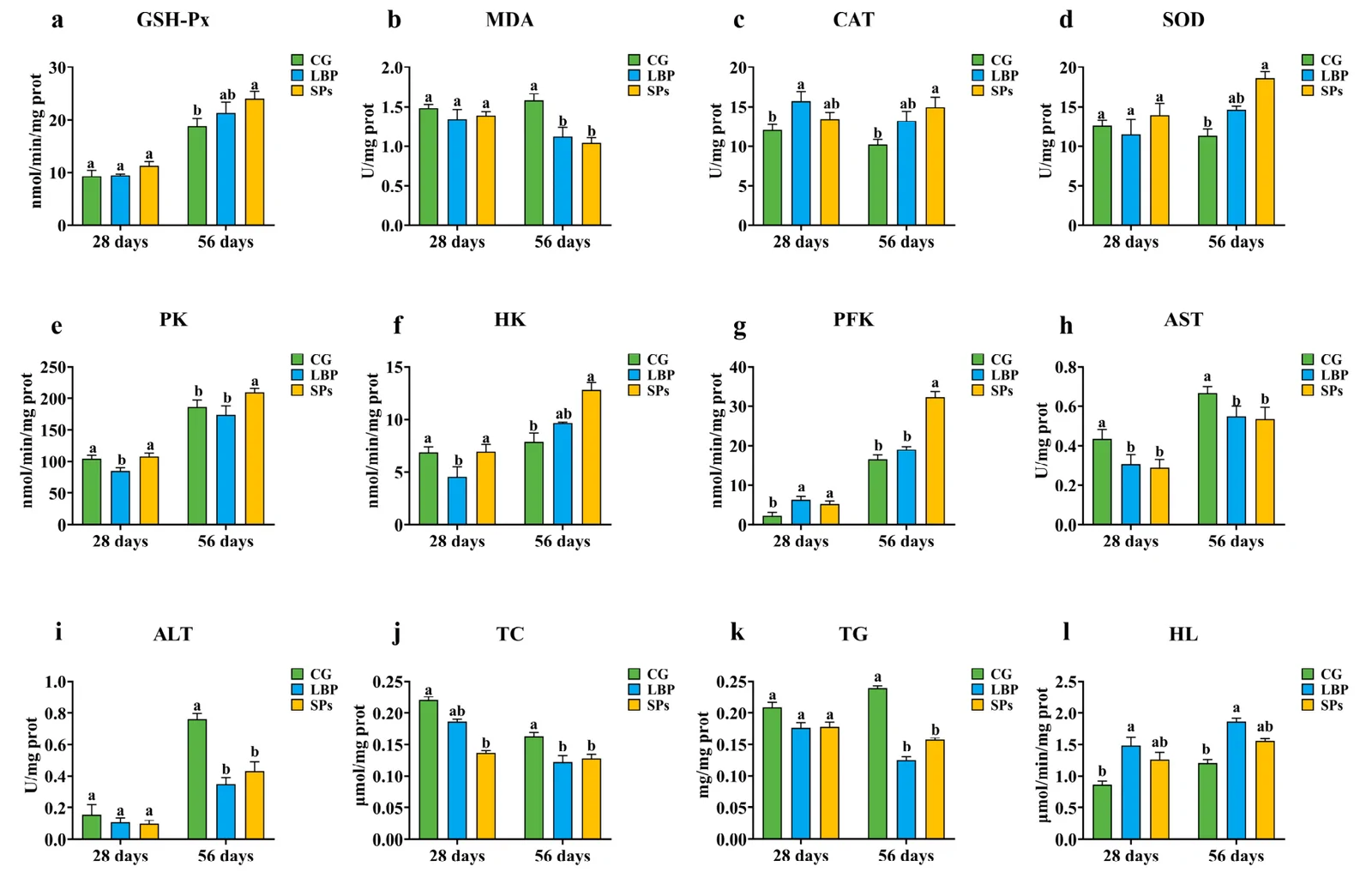
Effects of polysaccharides on the specific activities of enzymes related to antioxidant indicators in rainbow trout: (**a**) GSH-Px; (**b**) MDA; (**c**) CAT; (**d**) SOD. Effects of polysaccharides on the specific activities of glycolytic enzymes in rainbow trout: (**e**) PK; (**f**) HK; (**g**) PFK. Effects of polysaccharides on the specific activities of liver function-related enzymes in rainbow trout: (**h**) AST; (**i**) ALT. Effects of polysaccharides on the activities of lipid metabolism-related enzymes in rainbow trout: (**j**) TC; (**k**) TG; (**l**) HL. Note: Different superscripted values (mean ± SEM) in the same row indicate significant differences among different groups within the same time period (*p* < 0.05).

Oxidative stress indicators (GSH-Px, MDA, CAT, SOD, Figure 3a–d): At day 28, the specific activity of CAT in the LBP group was significantly higher than in the CG and SPs groups, reaching 15.7 U/mg protein (*p* < 0.05). At day 56, GSH-Px, CAT and SOD activities in both the LBP and SP groups were significantly higher than in the CG (*p* < 0.05). MDA content was significantly lower, being 1.12 and 1.04 U/mg prot respectively (*p* < 0.05), with the SPs group showing significantly better effects than the LBP group.

Glycolytic enzymes (PK, HK, PFK, Figure 3e–g): At day 28, the specific activities of PK and HK showed no significant difference between the CG and SPs groups, but both were significantly higher than in the LBP group (*p* < 0.05). PFK activity was significantly higher in both the LBP and SP groups than in the CG, being 6.33 and 5.31 nmol/min/mg prot respectively (*p* < 0.05). At day 56, PK, HK, and PFK activities in the SPs group were significantly higher than in the CG and LBP groups, being 209.77, 12.81 and 30.26 nmol/min/mg prot respectively (*p* < 0.05).

Liver function and lipid metabolism indicators (AST, ALT, TC, TG, HL, Figure 3h–l): At day 28, the specific activity of AST in the LBP and SPs groups was significantly lower than in the CG (*p* < 0.05). TC content in the SPs group was significantly lower than in the CG, reaching 15.7 μmol/mg prot (*p* < 0.05), with a decreasing trend also in the LBP group. HL activity in the LBP group was significantly higher than in the CG and SPs groups, reaching 1.49 μmol/min/mg prot (*p* < 0.05). At day 56, the specific activities of AST and ALT in the LBP and SPs groups were significantly lower than in the CG (*p* < 0.05). TC and TG contents were also significantly lower in both treatment groups than in the CG (*p* < 0.05). HL activity in the LBP group remained significantly higher than in the SPs and CG, reaching 1.86 μmol/min/mg prot (*p* < 0.05).

### 3.5. Gene Expression

**Figure 4:**
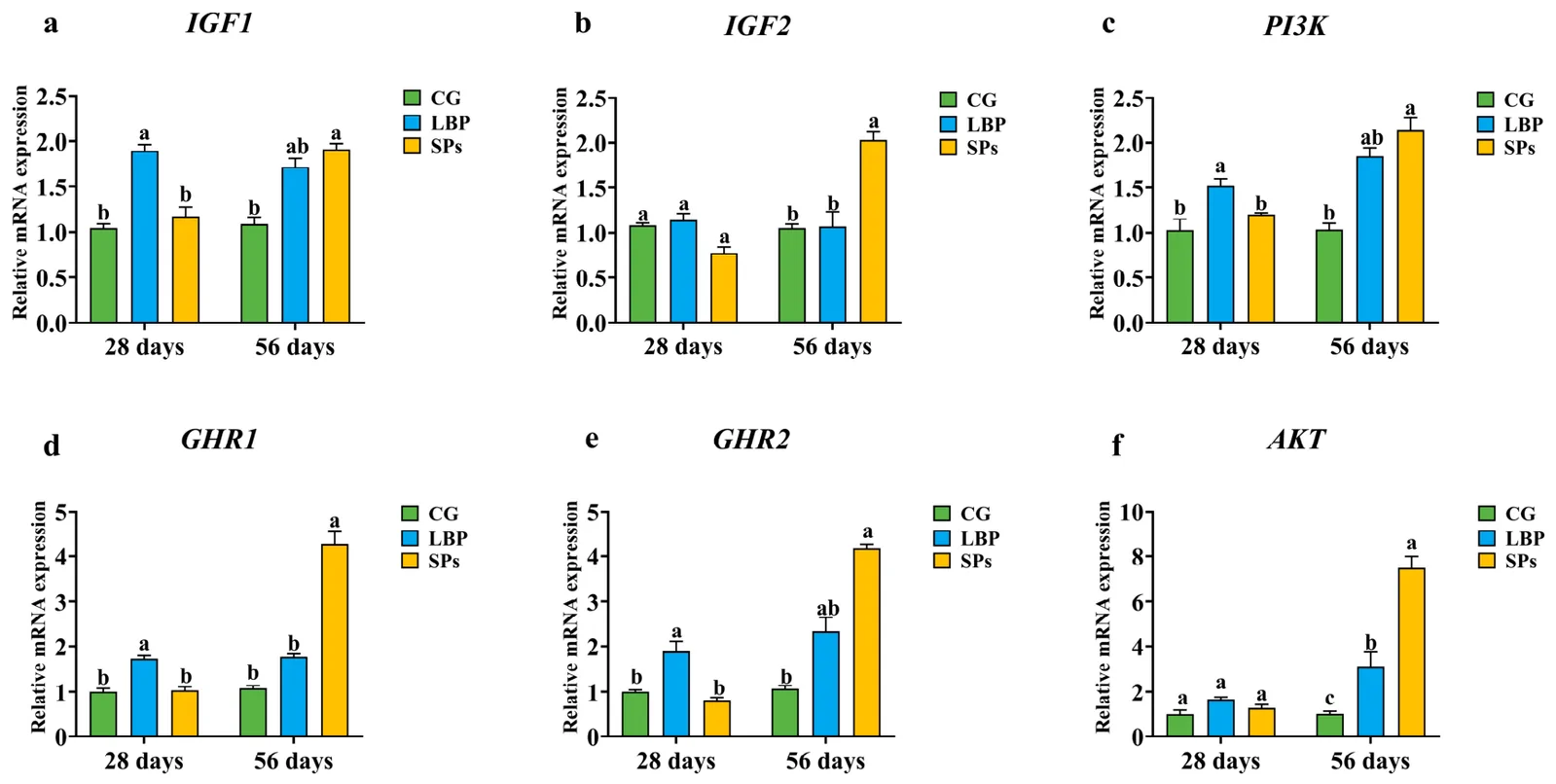
Effects of polysaccharides on the expression of growth-related genes in rainbow trout: (**a**) *IGF1*; (**b**) *IGF2*; (**c**) *PI3K*; (**d**) *GHR1*; (**e**) *GHR2*; (**f**) *AKT*. Note: Different superscripted values (mean ± SEM) in the same row indicate significant differences among different groups within the same time period (*p* < 0.05).

At day 28, mRNA expression of *IGF1*, *GHR1* and *GHR2* in the LBP group was significantly higher than in the CG (*p* < 0.05), and the expression of *PI3K* was significantly upregulated by 1.52-fold (*p* < 0.05; Figure 4c). At day 56, the SPs group showed significantly higher mRNA expression of *IGF1*, *IGF2*, *GHR1* and *GHR2* than both the CG and LBP groups (*p* < 0.05; Figure 4a,b,d,e). The expression levels of *AKT* were significantly higher than those in the CG by 1.86-fold and 2.14-fold (*p* < 0.05; Figure 4f).

**Figure 5:**
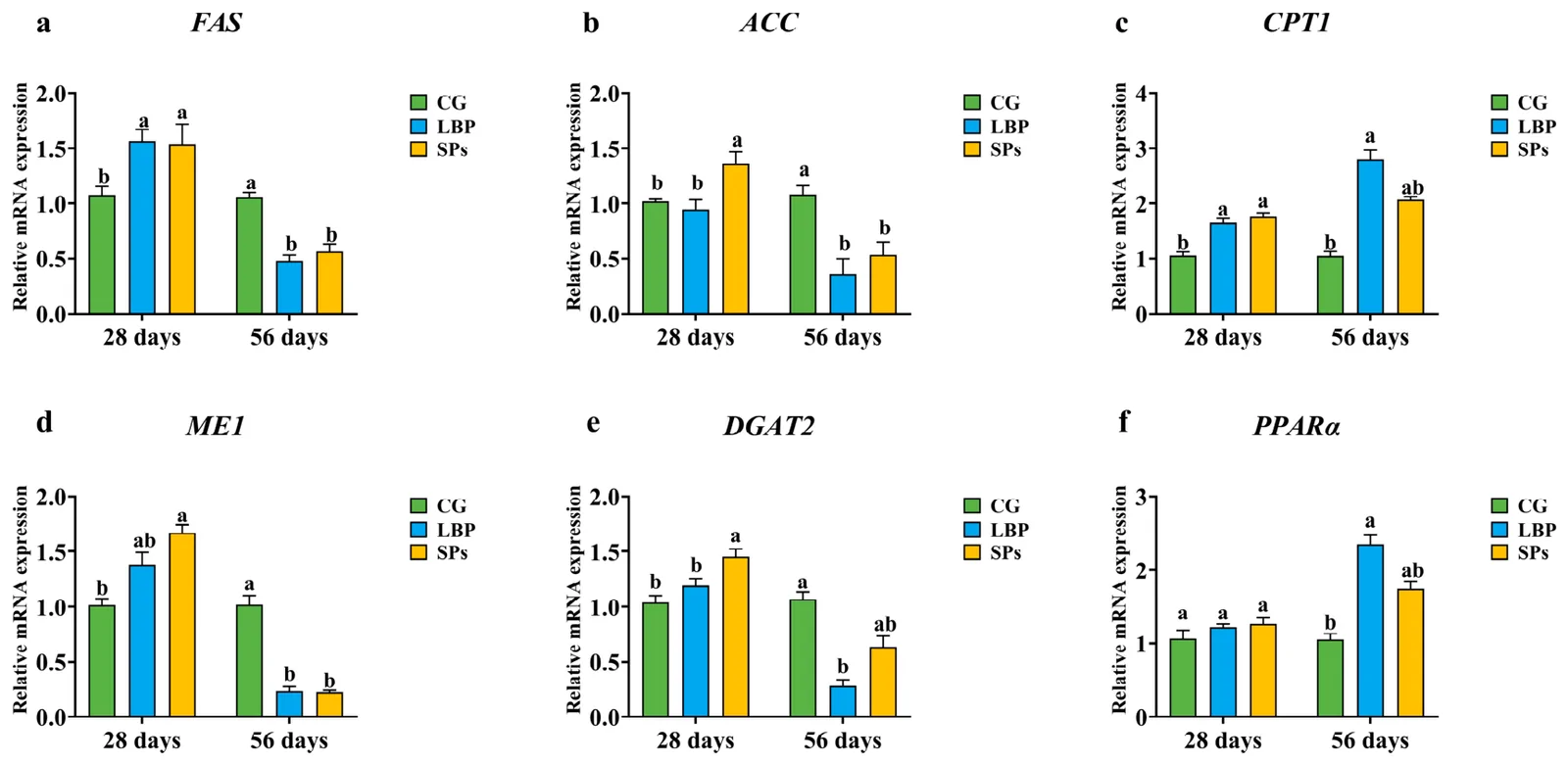
Effects of polysaccharides on the expression of lipid metabolism-related genes in rainbow trout: (**a**) *FAS*; (**b**) *ACC*; (**c**) *CPT1*; (**d**) *ME1*; (**e**) *DGAT2*; (**f**) *PPARα*. Note: Different superscripted values (mean ± SEM) in the same row indicate significant differences among different groups within the same time period (*p* < 0.05).

At day 28, mRNA expression of *FAS*, *ACC*, *ME1*, *CPT1* and *DGAT2* in the LBP and SPs groups was significantly higher than in the CG (*p* < 0.05; Figure 5a–e). At day 56, *CPT1* and *PPARα* expression in both treatment groups was significantly higher than in the CG, with LBP increasing them by 2.8-fold and 2.35-fold respectively (*p* < 0.05; Figure 5c,f), while *FAS*, *ACC*, *ME1* and *DGAT2* expression was significantly lower than in the CG (*p* < 0.05), with even lower levels in the LBP group.

**Figure 6:**
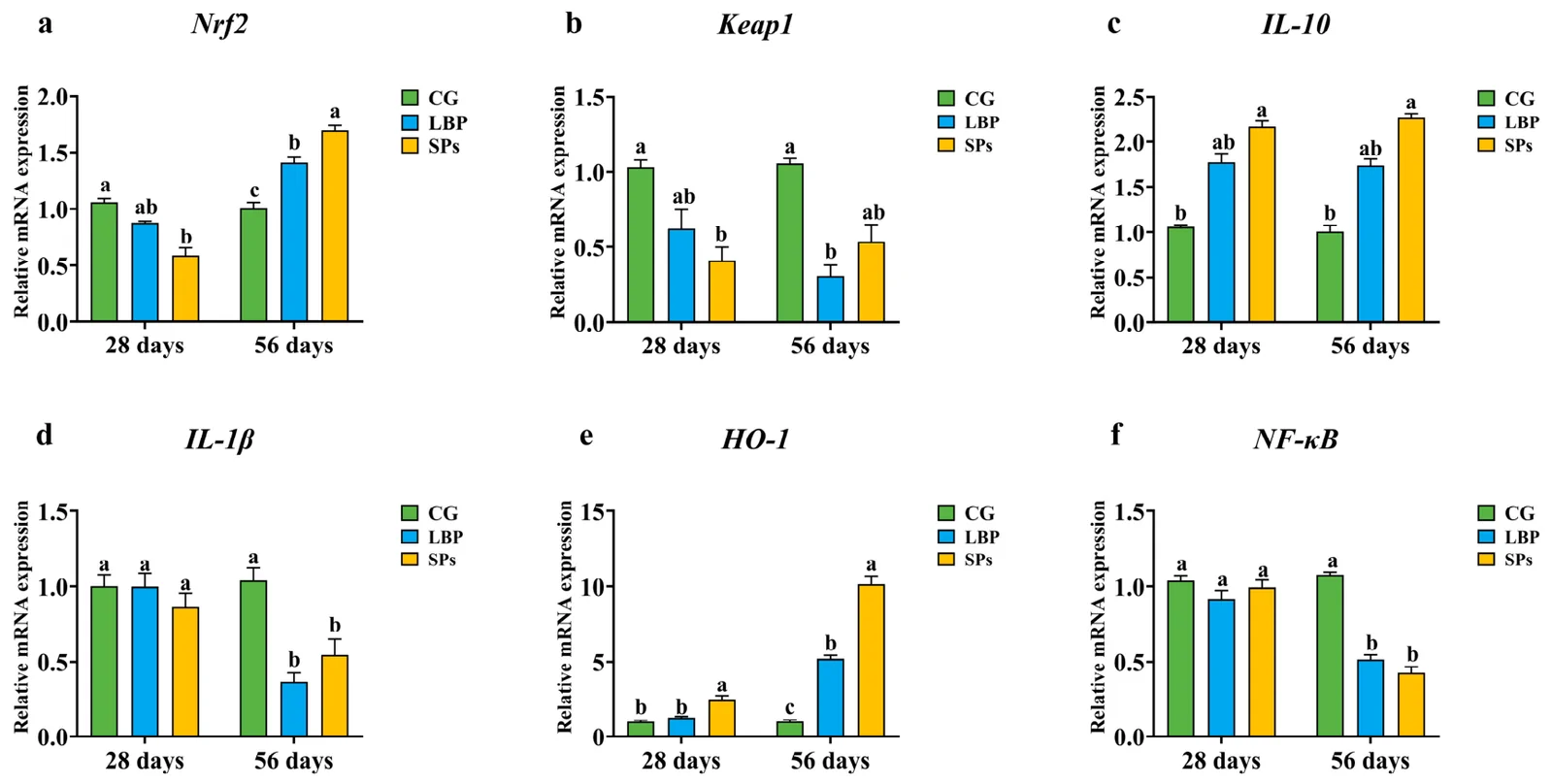
Effects of polysaccharides on the expression of immune-related genes in rainbow trout (**a**) *Nrf2*; (**b**) *Keap1*; (**c**) *IL-10*; (**d**) *IL-1β*; (**e**) *HO-1*; (**f**) *NF-κB*. Note: Different superscripted values (mean ± SEM) in the same row indicate significant differences among different groups within the same time period (*p* < 0.05).

At day 28, *Nrf2* and *Keap1* mRNA expression in the CG was significantly higher than in the LBP and SPs groups (*p* < 0.05; Figure 6a,b), while *HO-1* mRNA expression in the SPs group was significantly higher than in the CG and LBP groups, increasing by 2.48-fold (*p* < 0.05; Figure 6e). *IL-10* expression in the SPs group was significantly higher than in the CG, increasing by 2.16-fold (*p* < 0.05; Figure 6c). At day 56, *Nrf2* expression in the LBP and SPs groups was significantly higher than in the CG, increasing by 1.41-fold and 1.70-fold respectively (*p* < 0.05), and *Keap1* expression was significantly lower (*p* < 0.05). *HO-1* expression in both treatment groups was significantly increased relative to the CG, increasing by 2.20-fold and 10.14-fold respectively (*p* < 0.05), with *Nrf2* and *HO-1* expression in the SPs group significantly higher than in the LBP group (*p* < 0.05). *IL-10* expression in the SPs group remained significantly higher than in the CG, increasing by 2.27-fold (*p* < 0.05), while *IL-1β* expression was significantly lower (*p* < 0.05; Figure 6d). *NF-κB* expression in both the LBP and SPs groups was significantly lower than in the CG (*p* < 0.05; Figure 6f).

### 3.6. Intestinal Microbiota Changes

**Figure 7:**
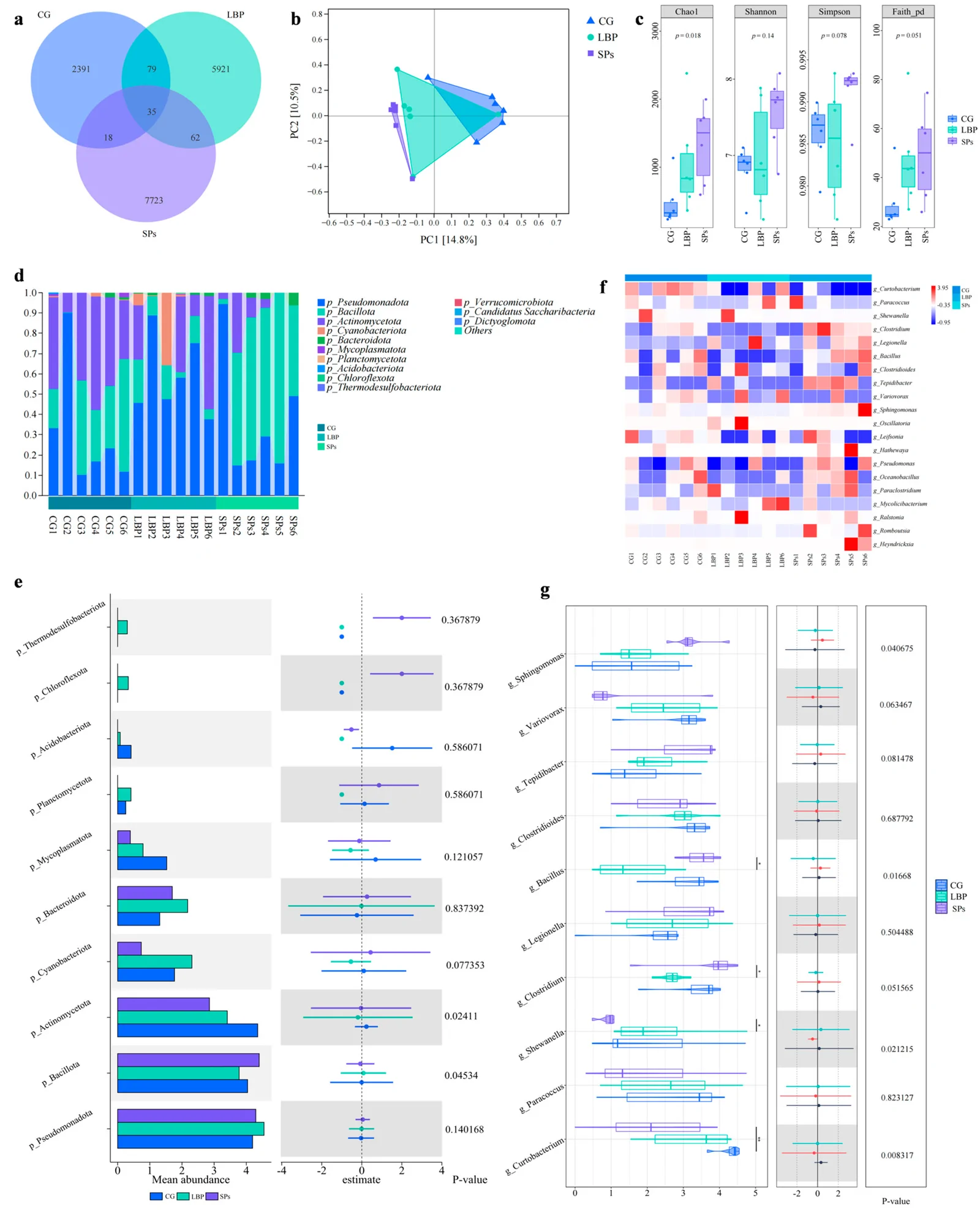
Effects of polysaccharides on intestinal microbiota diversity and microbiota composition in rainbow trout. (**a**) OTU quantity; (**b**) β diversity analysis–PCoA analysis; (**c**) α diversity analysis–Chao1 index, Shannon index, Simpson index, Faith_pd index; (**d**) relative abundance of the bacterial phylum; (**e**) heatmap visualizes the abundance of intestinal microbiota at the genus level; (**f**) Kruskal–Wallis test bar plot shows the relative abundance at the phylum level, “*” indicates *p* < 0.05, “**” indicates *p* < 0.01; (**g**) Kruskal–Wallis test bar plot shows the relative abundance at the genus level, the middle forest plot displays effect sizes of pairwise comparisons (LBP vs CG, red; SPs vs CG, teal; SPs vs LBP, dark blue), and the right panel presents corresponding *p*–values. “*” indicates *p* < 0.05, “**” indicates *p* < 0.01.

To explore polysaccharide regulation of intestinal microbiota, 16S rRNA sequencing was performed on CG, LBP, and SPs groups. Venn diagram (Figure 7a), OTU counts were 2391 (CG), 5921 (LBP), and 7723 (SPs), with 35 unique OTUs across groups, indicating high compositional specificity. PCoA (Figure 7b) showed that CG samples separated significantly from LBP and SPs; CG was highly clustered, while LBP and SPs were more dispersed, with SPs showing a more prominent regulatory effect. α diversity (Figure 7c) demonstrated that compared with CG, SPs had a significantly increased Chao1 index (*p* = 0.018) and a rising trend in Faith_pd (*p* = 0.051), indicating enhanced richness and phylogenetic diversity. Phylum-level composition (Figure 7d) showed that core phyla were *Pseudomonadota*, *Bacillota* and *Actinobacteriota*. Difference analysis (Figure 7e) showed that *Bacillota* abundance in SPs was significantly higher than in CG and LBP (*p* < 0.05); *Actinobacteriota* was highest in CG and decreased in LBP and SPs. Genus-level heatmap (Figure 7f) demonstrates that CG had higher *Cardobacteriaceae* and *Fusobacterium*; LBP and SPs had increased *Shewanella*, *Lactobacillus* and *Bacillus*. Genus-level difference analysis (Figure 7g) showed that SPs showed significantly increased *Sphingomonas* (*p* = 0.040675) and decreased *Curtobacterium* (*p* = 0.008317). These results indicate that LBP and SPs regulate specific genus abundance at the phylum level and reshape microbiota composition, with SPs showing more significant regulation of key genera.

### 3.7. Intestinal Contents Metabolites

**Figure 8:**
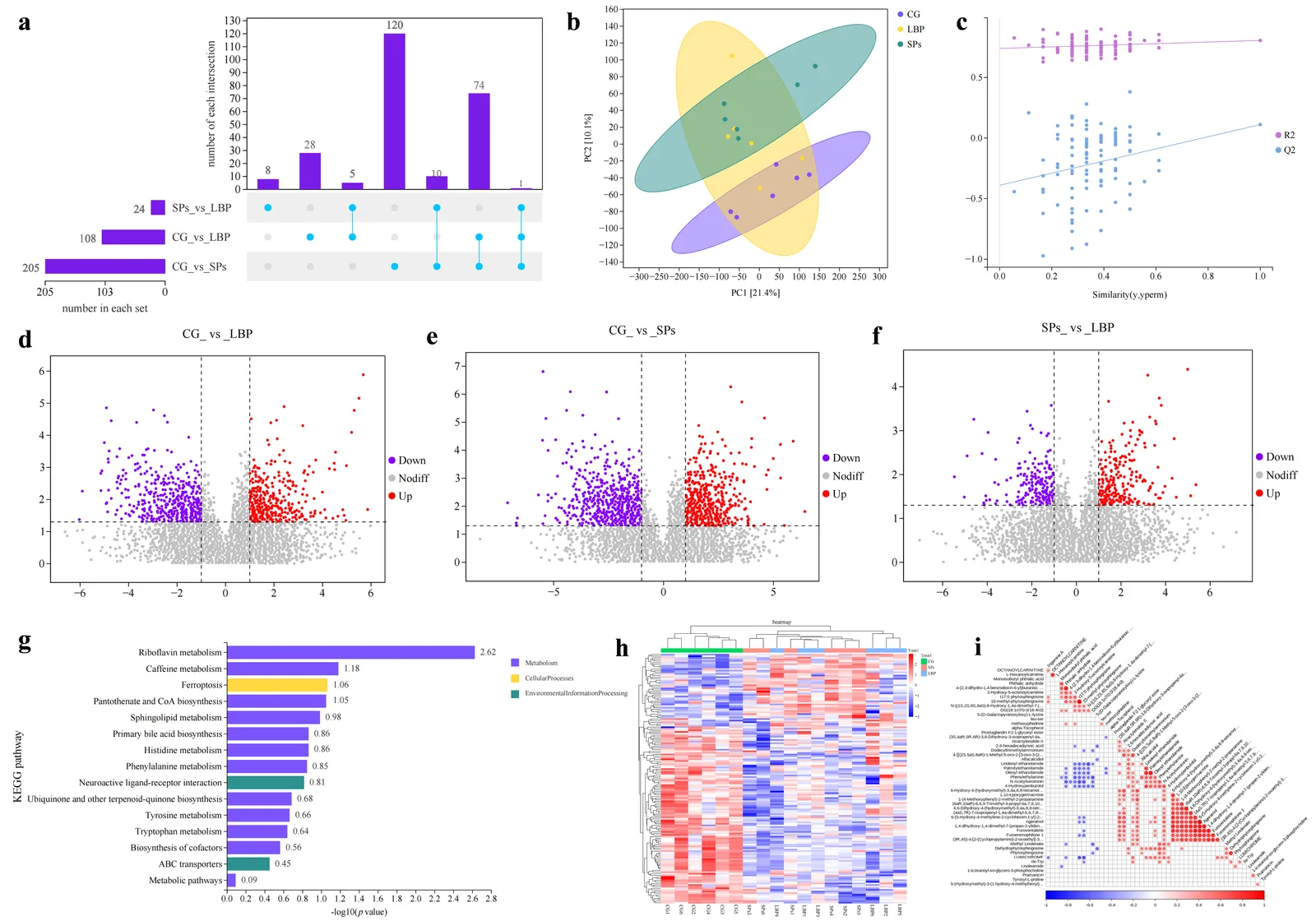
Effects of polysaccharides on metabolites from intestinal contents of rainbow trout: (**a**) upset diagram analysis of different metabolites between groups; (**b**) multivariate statistical PCA analysis chart of differential metabolites; (**c**) permutation test plot of the content of metabolites in PLS-DA; (**d**) volcano plot analysis of differentially expressed metabolites in CG_ vs. _LBP; (**e**) volcano plot analysis of differentially expressed metabolites in CG_ vs. _SPs; (**f**) volcano plot analysis of differentially expressed metabolites in SPs_ vs. _LBP; (**g**) KEGG pathway in differentially expressed metabolites; (**h**) clustering heatmap analysis of differential substances, Red: High abundance; Blue: Low abundance; Top color bar: Sample grouping CG/LBP/SPs; (**i**) quantitative analysis of differential metabolites, Red: Positive correlation; Blue: Negative correlation; White: No significant correlation.

To investigate metabolic profile differences among CG, LBP, and SPs’ intestinal contents, non-targeted metabolomics was performed. Common metabolites: 205 between CG and LBP, 108 between CG and SPs, and 24 between LBP and SPs, indicating significant specificity (Figure 8a). PCoA (Figure 8b) showed significant separation of CG from LBP and SPs, with distinct clustering among the three groups, indicating that LBP and SPs significantly altered metabolic profiles. PLS-DA (Figure 8c) confirmed overall metabolite distribution similarities and differences, providing a basis for differential metabolite identification. Volcano plots (Figure 8d–f) demonstrate that both LBP and SPs can induce significant changes in molecular expression, and the regulatory effects are not exactly the same, with specific differentially expressed molecules present. KEGG enrichment (Figure 8g) showed that differential metabolites were mainly enriched in riboflavin metabolism, caffeine metabolism, ubiquinone and terpenoid-quinone biosynthesis, as well as lipid metabolism, amino acid metabolism, and environmental information processing, indicating that LBP and SPs affect intestinal microecological stability by regulating host metabolic pathways. The hierarchical clustering heatmap (Figure 8h) showed clear distinction of metabolite expression patterns among the three groups, with specific clustering features between LBP and SPs, further verifying significant metabolic profile differences. The correlation heatmap (Figure 8i) revealed co-expression and interactions among differential metabolites, with some showing significant positive/negative correlations, forming the core molecular network regulated by LBP and SPs on intestinal metabolism.

### 3.8. Survivability After Bacterial Challenge

**Figure 9:**
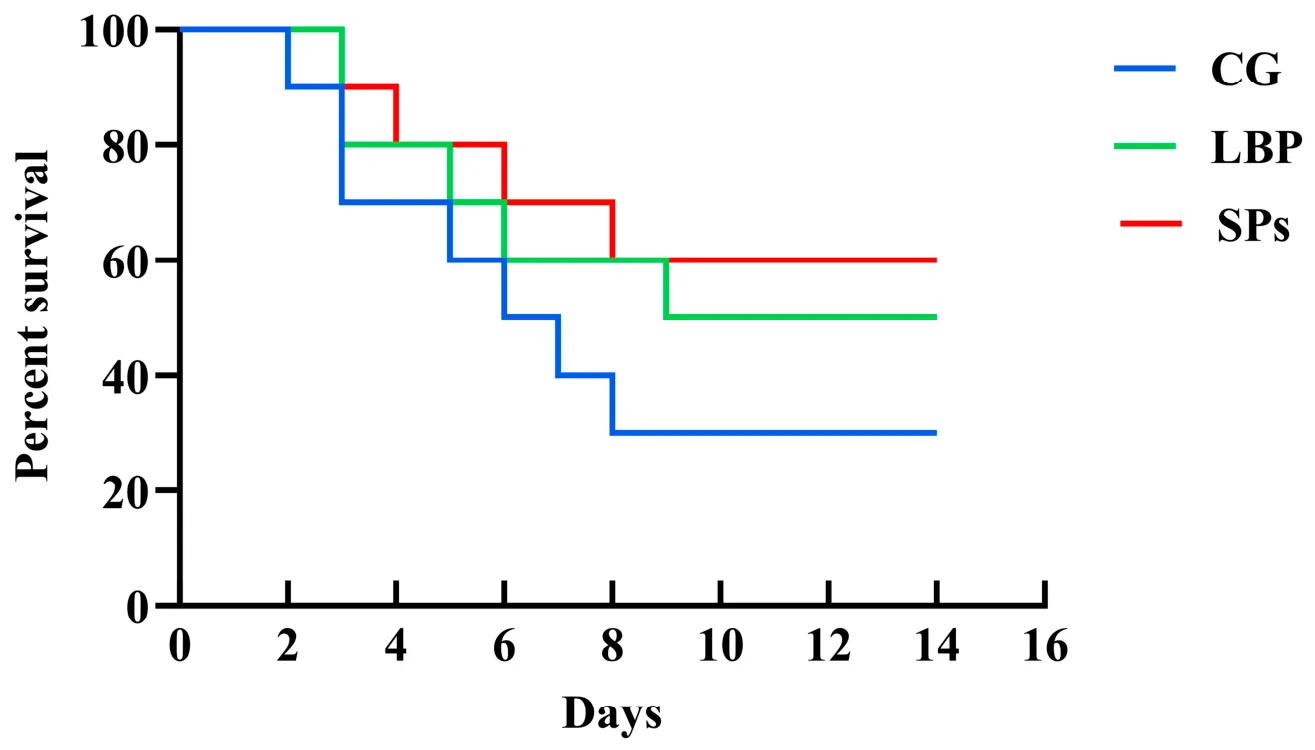
Percent survival of each group of fish 14 days after bacterial infection with *Aeromonas salmonicida*.

Following the *Aeromonas salmonicida* challenge in rainbow trout (Figure 9), the CG showed a rapid survival decline to 30% by day 8. In contrast, the LBP and SPs groups had substantially higher survival rates than the CG, with the SPs group slightly higher than the LBP group, remaining at approximately 60%.

## 4. Discussion

### 4.1. Growth Performance and Morphological Indicators

In recent years, polysaccharides have been widely used as additives in aquatic feed. Plant polysaccharides can enhance the growth performance and feed utilization of aquatic animals by stimulating the secretion of growth-related hormones, enhancing glucose and lipid metabolism, and increasing amino acid and protein synthesis [57,58,59]. Previous studies have shown that in a dose range of 1–2 g/kg of feed, various plant polysaccharides can effectively promote the growth performance, enhance the immune ability, and regulate the metabolic function of aquatic animals [11,23,32]. For instance, dietary supplementation with 1.5 g/kg of LBP significantly enhances the non-specific immunity of *Oreochromis niloticus* and reduces the rate of apoptosis, promoting growth and development [11,23,32]. Additionally, studies have also demonstrated that dietary supplementation with 1.5 g/kg of sulfated polysaccharides extracted from marine macroalgae can significantly improve the growth rate of *Oncorhynchus mykiss*, as well as enhance the immune system and pressure resistance [60], which is consistent with the results of this study. This study shows that adding LBP or SPs to the feed can substantially increase the WGR and SGR of rainbow trout fry and reduce the FCR. Among them, the effect of SPs was more significant. The growth promotion observed here underscores the potential of polysaccharides like LBP and SPs to serve as effective green feed additives. This may be related to the healthier condition of the fish, and more nutritional reserves are used for nutrient deposition. Furthermore, both LBP and SPs have reduced the VSI and HSI of rainbow trout fry to varying degrees. The effect of LBP is superior to that of SPs. Moreover, the AFP in the LBP group was significantly lower than that in the CG and the SPs group. This result is consistent with previous research findings, such as adding LBP to the diet of *Micropterus salmoides* [61], which can reduce VSI and HSI. Thus, supplementing feed with active polysaccharides appears to reduce lipid accumulation by promoting its breakdown, thereby improving overall body composition and visceral health in fish.

### 4.2. Composition of Amino Acids and Fatty Acids

Dietary supplementation with LBP and SPs was found to influence, to different extents, the muscle amino acid and fatty acid profiles of rainbow trout. The increase in amino acid content in muscles may be related to protein conservation mediated by lipid metabolism. This effect is achieved by enhancing fat breakdown, which reduces protein breakdown and thereby increases protein deposition and improves protein utilization [21]. At the same time, polyunsaturated fatty acids, as an important energy source substance for fish bodies, are easily decomposed to provide energy supply, providing sufficient ATP for the growth (cell proliferation and weight increase) of fish, thereby promoting growth [62]. In this study, the contents of various essential amino acids (Met, Arg) in the LBP and SPs groups were significantly increased, and the content in the SPs group was slightly higher than that in the LBP group. Met, as a sulfur-containing amino acid, is a substrate for protein synthesis [63], while Arg regulates vascular dilation through the NO pathway and participates in the regulation of protein deposition [64,65]. Such a mechanism helps to increase protein deposition and promotes the growth of fish. This result is consistent with previous studies on hybrid grouper (*Epinephelus lanceolatus*♂ × *E. fuscoguttatus*♀) [66] and *Oncorhynchus mykiss* [60] on the amino acid and fatty acid contents in muscles after dietary supplementation with LBP and seaweed sulfate polysaccharides. Therefore, polysaccharide substances may promote lipid breakdown to provide energy, reduce the consumption of protein as an energy source, and thereby promote protein accumulation in tissues, ultimately benefiting the growth of aquatic animals and improving the nutritional quality of fish meat [66].

### 4.3. Glycolysis, Lipid Metabolism and Antioxidant Capacity

Acting as the central organ for substance metabolism, the liver serves as the primary site for both glucose and lipid energy metabolism, while also functioning as a key hub for lipid synthesis, breakdown, and transport [67,68]. *Enteromorpha prolifera* polysaccharides can influence glucose metabolism by regulating key enzymes in glycolysis and the tricarboxylic acid cycle (TCA cycle), such as HK, PK, and PFK [69]. The results of this study indicate that the SPs group significantly increased the activities of key glycolytic enzymes PK, HK, and PFK, which is consistent with the research results on the dietary supplementation effects of mannan oligosaccharides on *Oreochromis niloticus* [70] and *corbicula fluminea* polysaccharides on *Monopterus albu* [71]. This suggests that SPs have the potential to activate glycolysis and optimize energy supply, which may enhance the efficiency of glycolytic energy supply and provide sufficient energy support for cell proliferation and protein deposition, thereby synergistically promoting the growth of rainbow trout. At the same time, polysaccharides have been proven to have the effect of reducing fat formation. They can inhibit fat synthesis by affecting the activity of enzymes related to fat formation, thereby regulating abnormal lipid metabolism. For example, research has demonstrated that pomelo fruit-derived polysaccharides effectively reduce hepatic lipid deposition in hybrid grouper (*Epinephelus lanceolatus*♂ × *Epinephelus fuscoguttatus*♀) [72]. Consistent with this, both LBP and SPs significantly reduced the TC and triglyceride TG levels in the liver, and LBP further demonstrated the ability to regulate lipid metabolism, achieving this effect by inhibiting HL activity, thereby reducing lipid deposition and maintaining the health of metabolic organs.

As the primary organ for detoxification, the liver also plays this essential role [73]. Oxidative stress induced by excessive lipid accumulation in fish results in the generation of abundant reactive oxygen species [58]. If these free radicals are not removed in time, the organism will be at risk of oxidative damage. Fish possess an efficient antioxidant enzyme system: SOD decomposes superoxide anion into O_2_ and H_2_O_2_, while CAT and GSH-Px further convert H_2_O_2_ into H_2_O and O_2_, preventing oxidative damage [66]. As a lipid peroxidation biomarker, MDA indirectly indicates the degree of tissue oxidative damage by reflecting its rate and intensity [74]. In this study, LBP and SPs supplementation elevated the activities of antioxidant enzymes (SOD, GSH-Px, CAT) and lowered MDA levels in rainbow trout. The effect of the SPs group was better than that of the LBP group. Consistent with previous research results, poria cocos and astragalus polysaccharides can also substantially increase the antioxidant enzyme activity of *Acipenser dabryanus* [75] and reduce the MDA level. This further indicates that polysaccharides have the ability to eliminate free radicals and alleviate lipid peroxidation damage. AST and ALT activity increases usually indicate an increase in amino acid conversion or metabolic waste in the tissue, which is an important sign of liver damage [76,77]. In this study, dietary supplementation with LBP and SPs substantially reduced hepatic AST and ALT activities, thereby improving liver function and contributing to the maintenance of overall health. The effect of the SPs group was better than that of the LBP group. Consistent with prior reports, astragalus polysaccharides and sea buckthorn polysaccharides have shown similar effects in reducing AST and ALT activities in *Channa argus* [78] and *Danio rerio* [79]. This indicates that dietary supplementation of polysaccharides can promote liver metabolic function and maintain the health of the organism.

### 4.4. Gene Expression Related to Growth, Lipid Metabolism, and Immunity

Insulin-like growth factors (IGFs) are recognized as key mediators through which the body responds to growth hormone (GH) stimulation, orchestrating most of the associated physiological effects. Previous studies have demonstrated that dietary supplementation with SPs enhances the expression of *IGF1* and *IGF2* in *Litopenaeus vannamei* [80], whereas LBP elevates *IGF1* expression in *Carassius auratus gibelio* [32]. The present study aligns with these findings, showing that SPs substantially upregulated the expression of growth hormone receptor genes (*GHR1* and *GHR2*) as well as *IGF1* and *IGF2*, while LBP specifically increased *IGF1* expression. These results suggest that both polysaccharides may modulate growth via distinct endocrine pathways, indicating their potential to activate the GH signaling cascade, thereby promoting protein synthesis and somatic cell proliferation [81]. The PI3K/AKT signaling pathway is a crucial intracellular pathway that regulates cell growth, proliferation, metabolism, survival, and migration [82]. The results of this study show that dietary supplementation with SPs significantly increased the expression of *PI3K* and *AKT*, which is associated with altered expression of PI3K/AKT pathway-related genes. These changes may be related to the synthesis of proteins, lipids, and nucleic acids, and may provide a material basis for cell division and growth; however, further protein-level and functional validation is needed to confirm whether the PI3K/AKT pathway is directly involved.

As the central organ for lipid metabolism, the liver participates in the digestion, absorption, synthesis, breakdown, and transport of lipids [83]. Key enzymes involved in lipogenesis, *FAS*, *ACC*, *DGAT2* and *ME1* are critical for lipid biosynthesis, whereas *PPARα* and *CPT1* are pivotal regulators of fatty acid oxidation. Prior research has indicated that polysaccharide supplementation can downregulate hepatic lipogenic gene expression, suggesting an inhibitory effect on fat accumulation [61]. *ME1* and *FAS* are key enzymes in adipogenesis, providing NADPH for fatty acid synthesis. As an electron donor, NADPH participates in the reductive biosynthesis of fatty acids, steroids, and DNA. *CPT1* is a critical enzyme in lipolytic metabolism [84]. *CPT1*, regulated by PPARα, catalyzes the conversion of lipoyl CoA to lipoyl carnitine, thereby initiating mitochondrial β-oxidation of long-chain fatty acids [85]. In this study, both LBP and SPs substantially suppressed the expression of *FAS* and *ACC*, while enhancing the expression of *CPT1* and *PPARα*, indicating their dual capacity to inhibit lipogenesis and promote lipid catabolism, with LBP exhibiting a more pronounced effect. This observation is supported by previous studies on *Cyprinus carpio* [23], in which dietary tea polysaccharides and LBP were shown to improve the expression of genes related to lipid metabolism. Furthermore, oil red O staining revealed a reduction in hepatic lipid droplets in both polysaccharide-supplemented groups, particularly in the LBP group, providing histological evidence supporting the beneficial impact of polysaccharides as feed additives on hepatic lipid deposition.

Immune regulation relies on the coordinated expression of immune-related genes [86]. Upon exposure to external stimuli, inflammatory responses are initiated, and immune homeostasis is maintained through the balanced expression of *IL-1β* and *IL-10* [87]. In this study, both LBP and SPs substantially enhanced *IL-10* expression while suppressing *IL-1β* levels, indicating their roles in maintaining immune balance and mitigating excessive inflammation. These findings are consistent with prior studies showing that ginger polysaccharides and yeast polysaccharides modulate inflammatory gene expression in *Carassius auratus* [88] and *Micropterus salmoides* [89]. Active polysaccharides have been reported to alleviate inflammatory responses via *NF-κB* inhibition. In the present study, *NF-κB* gene expression was downregulated in the SPs group. At the same time, SPs more potently upregulated *HO-1*, implying that their hepatoprotection may stem from activating the Nrf2/HO-1 pathway, which consequently bolsters cellular antioxidant defenses [90]. In combination with the results of the pathogen attack, in the LBP and SPs groups, the survival rate of rainbow trout was significantly higher than that in the CG, with the SPs group having the highest survival rate. This indicates that it has a stronger ability to resist diseases. This further proves the strengthening effect of polysaccharides on immunity and disease resistance.

### 4.5. Intestinal Biological Barrier and Metabolomics

Plant polysaccharides optimize the structure of the intestinal microbiota, promote the proliferation of beneficial bacteria, provide additional nutritional support for growth, and enhance feed utilization efficiency [64]. In this study, both LBP and SPs significantly altered the composition and diversity of the intestinal microbiota of rainbow trout. Among them, the Chao1 index of the SPs group significantly increased, the richness of the microbiota improved, and *Clostridium* and other beneficial bacteria were significantly increased at the genus level, while the relative abundance of *Curtobacterium*, which dominated in the CG, decreased. This achieved the targeted proliferation of beneficial bacteria and the effective inhibition of harmful bacteria in the intestinal tract. As one of the core beneficial bacteria in the intestinal tract, *Clostridium* can directly participate in the synthesis of nutrients such as amino acids and short-chain fatty acids (SCFAs), providing additional nutritional substrates for growth and compensating for the deficiency of nutrients in the feed [91]. At the same time, the optimization of the intestinal microbiota mediated by polysaccharides significantly improved the intestinal morphology. The VH, VW, CD and MT in the SPs group were significantly higher than those in the CG and LBP groups. The intestinal mucosal absorption area significantly increased, and the promotion effect of beneficial bacteria on the secretion of digestive enzymes enhanced the digestibility and absorption efficiency of proteins, fats, and other nutrients in the feed, reducing nutrient loss [92]. In addition, the amino acids synthesized by the proliferation of beneficial bacteria can be directly absorbed and utilized by the intestinal tract, providing raw materials for cell proliferation and muscle growth [62].

Systematic remodeling of the intestinal metabolic profile acts as the functional hub for plant polysaccharides in regulating systemic metabolic homeostasis [93]. The analysis of differential metabolites revealed that the SPs group exhibited 205 differential metabolites compared to the control group, significantly more than the CG and the LBP group. These included various amino acids and short-chain fatty acid precursors. This metabolic feature is highly synchronized with the changes in the microbiota structure. In the study of *Carassius auratus* [94], it was also confirmed that polysaccharides can improve meat quality by regulating the intestinal microbiota and amino acid metabolic networks. At the same time, the metabolic signature shaped by the intestinal microbiota provides important metabolic signals for immune regulation, and the upregulated amino acids, short-chain fatty acids and other metabolites can be transported to the liver and the entire body through the liver-intestinal axis [95]. Among them, immune-related amino acids such as Arg provide material support for the synthesis of immune factors and the proliferation of immune cells [96], while short-chain fatty acids can regulate the expression of immune-related genes in the liver and achieve precise regulation of the inflammatory response, effectively alleviating chronic inflammation under high-density farming [97].

Based on the results of this study, SPs demonstrate better performance in promoting growth and enhancing immunity, while LBP shows superior efficacy in metabolic regulation improvement. Several possible reasons may account for these distinct effects. First, the two polysaccharides differ in their chemical structure and monosaccharide composition. SPs are sulfated polysaccharides rich in fucose and sulfate groups, which have been reported to interact with gut-associated immune cells [98] and to promote the proliferation of beneficial bacteria such as *Bacillus* and *Lactobacillus* [99], thereby enhancing nutrient absorption and immune defense. In contrast, LBP is a neutral heteropolysaccharide rich in arabinose, galactose, and glucose, which has been reported to regulate hepatic lipid metabolism through modulation of PPARα and its downstream target genes such as *CPT1*, *FAS*, and *ACC* [11]. Second, the two polysaccharides may be fermented differently by the gut microbiota, producing distinct profiles of short-chain fatty acids and metabolites that act on different host tissues [11,32]. Third, the observed differences may reflect differences in the bioavailability and tissue distribution of the two polysaccharides, with SPs acting more strongly on the intestinal mucosa and systemic immune system, and LBP acting more strongly on the liver [98].

However, this study also has several limitations that point to directions for future research. First, this study tested only a single supplementation level of LBP and SPs (1.5 g/kg), which does not allow assessment of dose–response relationships. Future studies will test graded concentrations to determine the optimal inclusion level and the effective concentration range of each polysaccharide. Second, the mechanistic interpretation in this study is based mainly on mRNA expression data. Therefore, the pathway-related findings are described as changes in pathway-related gene expression rather than confirmed pathway activation. Future studies will validate these findings at the protein level, including Western blot analysis of key pathway proteins and their phosphorylation status, nuclear translocation assays, and pathway inhibition or activation experiments, to determine whether the Keap1/Nrf2, PPARα, and PI3K/AKT pathways are directly involved. Third, the bacterial challenge experiment evaluated survival rate and clinical signs as endpoints. Future studies will extend these measurements to include bacterial load in liver, spleen, and kidney; histopathological lesion scoring; and immune parameters such as serum lysozyme activity, complement levels, and immunoglobulin M, in order to further elucidate the immune mechanisms underlying the improved survival observed in this study. Addressing these limitations in future work will help to confirm and extend the present findings and provide a stronger basis for the combined use of LBP and SPs as feed additives in rainbow trout aquaculture.

## 5. Conclusions

In conclusion, dietary LBP and SPs (each 1.5 g/kg) enhanced growth, metabolism, and immunity in juvenile rainbow trout through distinct contributions. SPs were more effective for growth, antioxidant capacity, immune function, and disease resistance, with increased gut microbiota richness and beneficial genera. LBP showed superior metabolic regulation, including reduced hepatic lipid accumulation, lower TC and TG, and altered PPARα pathway-related gene expression. These findings suggest complementary rather than identical mechanisms. LBP primarily targets hepatic lipid metabolism and intestinal health, whereas SPs mainly enhance antioxidant and immune defenses. This complementarity supports their combined use to improve growth, metabolism, immunity, and disease resistance in rainbow trout aquaculture. Future studies should compare individual versus combined supplementation to confirm additive or synergistic effects.

## Figures and Tables

**Table 1:** Quantitative real-time PCR primers.

Gene	Forward (5′→3′) Reverse (5′→3′)	Accession Number	Primer Efficiency (%)	Reference
*IL-1β* *	CTCTACCTGTCCTGCTCCAAA ATGTCCGTGCTGATGAACC	XM_036979104.1	94.00	[41]
*IL-10*	CGACTTTAAATCTCCCATCGAC GCATTGGACGATCTCTTTCTTC	AB118099.1	97.00	[41]
*Nrf2*	TGAGCTGCAGCAATGTCTGA GTTGGGCAATGGGTAGAAGC	NM_001124268.1	93.00	[42]
*Keap1* *	ACGACGTTGAGACTGACACC TCCCGTCATAACCTCCCAGT	XM_036949280.1	93.00	[43]
*HO-1*	CGCCTACACCCGTTACCTAG CTCTCCGCTGCTTAACCCAA	AF099079.2	91.00	[44]
*NF-κB*	CAGCGTCCTACCAGGCTAAAGAGAT GCTGTTCGATCCATCCGCACTAT	BX880658.3	98.00	[41]
*FAS* *	CCTCACCTCCAAAGACAGTCAG CGACGGCGACACAGGAATAA	XM_021576226.2	96.00	[45]
*ACC* *	TCATCAATGCCAAGGACCCC CGTCAGAGTCCAGGTTTGCT	XM_036990266.1	94.00	[45]
*DGAT2*	GGAACACCCCCAAACAAGGT TACTGCCCCAACACCACATC	LOC110533663	99.00	[46]
*ME1*	CCATTGGAGGGGCGTTTACT GGGGTTACTCAGGGCGAAAA	XM_021597955.1	95.00	[47]
*PPARα*	CACCATTCTAGATGCGCTGA GGCGTGAACTCCATAGTGGT	KP342260	93.00	[48]
*CPT1*	GCACTGCAAAGGAGACATCA GCTATCACCTTGGCAACCAT	AF327058	93.00	[49]
*GHR1*	TGGACACCCAGTGCTTGATG TCCCTGAAGCCAATGGTGAT	AB10083	97.00	[35]
*GHR2*	TCGGAACATTCCAGAACCTC GGTCATCCAGACCTTCGTGT	NM_001123594	101.00	[50]
*IGF1* *	ACTGTGCCCCTGCAAGTCT CTGTGCTGTCCTACGCTCTG	XM_021577177.2	95.00	[41]
*IGF2*	GAAGGTCAAGATGATGTCTTCG AGTTCTCCTCCACATAGCGTTT	NM_001124697.1	104.00	[51]
*PI3K* *	TGTTGAGGGTGAGGTGAAGCA AAACTGGGTAGGCGAGCGT	XM_021606760.2	92.00	[52]
*AKT* *	TGACGCCAAGGAGATAATGC GGTGTCTGTTTCCGAGGTGA	XM_021600591.2	98.00	[52]
*β-actin* *	GATGGGCCAGAAAGACAGCTA TCGTCCCAGTTGGTGACGAT	XM_036972332.1	99.00	[53]
*GAPDH* *	GAGGGTCTGATGAGCACAGTTC GATGACCTTGCCGACAGCC	XM_021623341.2	99.00	[54]

Note: *IL-1β*, Interleukin-1 beta; *IL-10*, Interleukin-10; *Nrf2*, Nuclear factor erythroid 2-related factor 2; *Keap1*, Kelch-like ECH-associated protein 1; *HO-1*, Heme oxygenase 1; *NF-κB*, Nuclear factor kappa B; *FAS*, Fatty acid synthase; *ACC*, Acetyl-CoA carboxylase; *DGAT2*, Diacylglycerol O-acyltransferase 2; *ME1*, Malic enzyme 1; *PPARα*, Peroxisome proliferator-activated receptor alpha; *CPT1*, Carnitine palmitoyltransferase 1; *GHR1*, Growth hormone receptor 1; *GHR2*, Growth hormone receptor 2; *IGF1*, Insulin-like growth factor 1; *IGF2*, Insulin-like growth factor 2; *PI3K*, Phosphoinositide 3-kinase; *AKT*, Protein kinase B; *β-actin*, Beta-actin; *GAPDH*, Glyceraldehyde-3-phosphate dehydrogenase. The symbols marked with “*” indicate the predicted sequences. These sequences were designed based on the reference sequences available at that time for primer design.

**Table 2:** Effects of polysaccharides on growth indices of rainbow trout.

Growth Index	CG	LBP	SPs
WGR (%)	297.17 ± 23.35 ^b^	312.14 ± 53.84 ^b^	414.22 ± 50.94 ^a^
SGR (%)	2.46 ± 0.16 ^b^	2.52 ± 0.23 ^b^	2.92 ± 0.18 ^a^
FCR	0.76 ± 0.09 ^a^	0.66 ± 0.11 ^ab^	0.42 ± 0.05 ^b^
VSI (%)	12.56 ± 3.17 ^a^	8.95 ± 2.36 ^b^	9.91 ± 1.00 ^ab^
HSI (%)	1.23 ± 0.24 ^a^	0.99 ± 0.23 ^ab^	0.92 ± 0.10 ^b^
CF (g/m^3^)	0.018 ± 0.001 ^a^	0.016 ± 0.002 ^a^	0.017 ± 0.002 ^a^
AFP (%)	1.63 ± 0.71 ^a^	1.54 ± 0.32 ^b^	1.63 1.63 ± 0.29 ^a^

Note: Values (mean ± SEM) in the same row with different superscripts are substantially different (*p* < 0.05).

**Table 3:** Effects of polysaccharide on intestinal tissue development of rainbow trout.

Item	CG	LBP	SPs
VH (µm)	545.38 ± 12.37 ^b^	632.63 ± 10.54 ^b^	947.99 ± 20.98 ^a^
VW (µm)	149.31 ± 11.31 ^b^	171.11 ± 16.11 ^ab^	190.35 ± 18.57 ^a^
CD (µm)	422.43 ± 22.19 ^b^	597.64 ± 18.07 ^b^	883.54 ± 27.21 ^a^
MT (µm)	109.22 ± 22.65 ^ab^	74.23 ± 15.67 ^b^	130.04 ± 19.23 ^a^

Note: Values (mean ± SEM) in the same row with different superscripts are substantially different (*p* < 0.05).

## Data Availability

Data will be made available on request.
